# Tetrabromocobalt Phthalocyanine-Functionalized Carbon Nanotubes as a High-Performance Anode for Lithium-Ion Batteries

**DOI:** 10.3390/nano15221713

**Published:** 2025-11-12

**Authors:** Keshavananda Prabhu Channabasavana Hundi Puttaningaiah

**Affiliations:** School of Chemical, Biological, and Battery Engineering, Gachon University, Seongnam-si 13120, Republic of Korea; golu12345@gachon.ac.kr

**Keywords:** lithium-ion batteries, tetrabromocobalt phthalocyanine, carbon nanotubes, hybrid materials, energy storage

## Abstract

The search for high-capacity, stable anode materials is crucial for advancing lithium-ion battery (LIB) technology. Although carbon nanotubes (CNTs) are known for their excellent electrical conductivity and mechanical strength, their practical capacity is still limited. This study presents an advanced anode design by molecular functionalizing both single-walled and multi-walled carbon nanotubes (SWCNTs and MWCNTs) with tetrabromocobalt phthalocyanine (CoPc), resulting in CoPc/SWCNT and CoPc/MWCNT hybrid materials. Metal phthalocyanines (MPcs) are recognized for their tunable and redox-active properties. In CoPc, the redox-active metal centers and π-conjugated structure are uniformly attached to the CNT surface through strong π-π interactions. This synergistic combination significantly boosts the lithium-ion (Li-ion) storage capacity by offering numerous coordination sites for Li-ions and enhancing charge transfer kinetics. Electrochemical analysis shows that the CoPc-SWCNT active anode electrode material shows an impressive reversible capacity of 1216 mAh g^−1^ after 100 cycles at a current density of 0.1 A g^−1^, substantially surpassing the capacities of pristine CoPc (327 mAh g^−1^) and a CoPc/MWCNT hybrid (488 mAh g^−1^). Furthermore, the CoPc/SWCNT anode exhibited exceptional rate capability and outstanding long-term cyclability. These results underscore the effectiveness of non-covalent functionalization with SWCNTs in enhancing the electrical conductivity, structural stability, and active site utilization of CoPc, positioning CoPc/SWCNT hybrids as a highly promising anode material for high-performance Li-ion storage.

## 1. Introduction

The relentless pursuit of advanced energy storage systems is fundamentally linked to the development of high-performance electrode materials. Lithium-ion batteries (LIBs), as the dominant power source for portable electronics and electric vehicles, face incessant demands for higher energy density, longer cycle life, and improved rate capability [1,2,3]. The commercial graphite anode, with a theoretical capacity limited to 372 mAh g^−1^, is increasingly becoming a bottleneck for next-generation LIBs [4]. Transition metal oxides come from significant volume expansion and silicon from serve mechanical instability during cycling. These all-critical limitations have spurred extensive research into alternative anode materials capable of hosting more lithium ions (Li-ion) through alloying, conversion, or novel storage mechanisms [5,6]. Among the numerous candidates, organometallic materials, particularly molecular complexes such as metallophthalocyanines (MPcs), have emerged as a promising class. Their appeal lies in their structural diversity, tunable redox properties, and potential for high theoretical capacities through multi-electron redox reactions at their conjugated macrocyclic rings and metal centers [7,8,9,10]. Tetrabromocobalt phthalocyanine (CoPc) is of significant interest due to the reversible redox activity of the Co^2+^/^+^ couple and its inherent π-conjugated system, which can facilitate electron transport [11,12]. However, the practical application of pristine CoPc is limited by its intrinsic poor electrical conductivity and tendency to dissolve in organic electrolytes, leading to rapid capacity fading and inferior rate performance [11,13,14].

A powerful strategy to mitigate these drawbacks is the integration of active organic molecules with conductive carbon nanomaterials. Carbon nanotubes (CNTs), with their exceptional electrical conductivity, mechanical strength, and high specific surface area, act as ideal hosts [15,16]. Non-covalent functionalization of CNTs with MPcs via π-π stacking interactions is especially attractive as it preserves the intrinsic electronic properties of both components while creating a stable, synergistic hybrid [17,18]. This architecture can enhance electron transfer, prevent the aggregation and dissolution of the active material, and provide a robust framework for withstanding volume changes during cycling. Although numerous studies have examined MPc-CNT composites, the specific impact of the CNT type, single-walled (SWCNT) versus multi-walled (MWCNT), on the electrochemical performance of substituted MPcs remains a complex issue that merits deeper investigation [12,19]. This molecular engineering approach aims to create a synergistic composite where the CNT network ensures efficient electron transport, while the MPc molecules provide abundant and accessible active sites for Li storage, thereby enhancing both capacity and ion diffusion kinetics for next-generation LIB anodes.

Herein, we report the synthesis of CoPc and its strategic integration with CNTs to create high-performance hybrid anode materials. A single-step condensation reaction was employed to synthesize the CoPc macrocycle, which was subsequently anchored onto SWCNTs and MWCNTs via a simple, non-covalent functionalization route in N-Methyl-2-pyrrolidone (NMP). The resulting hybrids, denoted as CoPc/SWCNT and CoPc/MWCNT, were systematically evaluated as anodes for LIBs. Electrochemical analysis reveals that the CoPc/SWCNT hybrid delivers an exceptional reversible capacity of 1216 mAh g^−1^ after 100 cycles at a current density of 0.1 A g^−1^, significantly outperforming both pristine CoPc (327 mAh g^−1^) and a CoPc/MWCNT hybrid (488 mAh g^−1^). Furthermore, the CoPc/SWCNT anode demonstrates remarkable rate capability and outstanding long-term cyclability. This work underscores the critical role of SWCNTs in maximizing the electrochemical properties of MPcs and presents a highly promising anode material strategy for advanced Li-ion storage.

## 2. Materials and Methods

### 2.1. Materials

4-bromophthalic anhydride (C_8_H_3_BrO_3_) (97%), cobalt (II) chloride hexahydrate (CoCl_2_·6H_2_O, 98%), ammonium chloride (NH_4_Cl, ≥99.5%), urea (CH_4_N_2_O, ≥99%), ammonium molybdate tetrahydrate ((NH_4_)_6_Mo_7_O_24_·4H_2_O, 99%), and nitrobenzene (C_6_H_5_NO_2_, 99%) were used for the MPcs synthesis. Single-walled carbon nanotubes (SWCNTs, >95% carbon basis) and multi-walled carbon nanotubes (MWCNTs, >95% carbon basis) were used as received from Nano Solution Company, Republic of Korea. N-Methyl-2-pyrrolidone (NMP, 99.5%) and ethanol (absolute) were used as solvents. All chemicals were purchased from Sigma-Aldrich and TCI (Republic of Korea) used without further purification.

### 2.2. Synthesis of CoPc

CoPc was synthesized via a single-step condensation reaction adapted from established methods [20,21,22,23]. In a typical synthesis, 4-bromophthalic anhydride (4 mmol), cobalt (II) chloride (1 mmol), urea (10 mmol), and ammonium chloride (6 mmol) were thoroughly ground together in a mortar and pestle. A catalytic amount of ammonium molybdate (ca. 10 mg) was added to the mixture. The homogeneous powder was transferred to a round-bottom flask and suspended in 20 mL of nitrobenzene. The reaction mixture was heated under reflux with vigorous stirring at 180–200 °C for 6–8 h. After cooling to room temperature, the resulting precipitate was collected by vacuum filtration. The crude product was washed sequentially with hot ethanol, hot water, and again with hot ethanol to remove unreacted precursors and the nitrobenzene solvent. The final dark green solid was dried overnight in a vacuum oven at 80 °C. Yield of CoPc: 87%.

### 2.3. Preparation of CoPc-CNT Hybrids

The CoPc-CNT hybrids were prepared via a solution-based non-covalent functionalization method. Briefly, 100 mg of the synthesized CoPc was dissolved in 20 mL of NMP by bath ultrasonication for 30 min to form a stable solution. Separately, 10 mg of either SWCNTs or MWCNTs were dispersed in 20 mL of NMP via probe sonication for 1 h to obtain a homogeneous black suspension. The CoPc solution was then added dropwise to the respective CNT dispersion under vigorous magnetic stirring. The resulting mixture was stirred at 80 °C for 12 h to facilitate π-π stacking interactions between the CoPc macrocycles and the CNT walls. The final product was collected by vacuum filtration through a polycarbonate membrane (0.22 μm pore size), washed repeatedly with ethanol and deionized water to remove any physically adsorbed molecules, and dried overnight in a vacuum oven at 60 °C. The resulting hybrids are denoted as CoPc/SWCNT and CoPc/MWCNT.

### 2.4. Electrochemical Measurements

The electrochemical performance was evaluated using CR2032-type coin cells assembled in an argon-filled glovebox. The working electrodes were prepared by mixing the active material (pristine CoPc, CoPc/SWCNT, or CoPc/MWCNT), conductive carbon black (Super P), and polyvinylidene fluoride (PVDF) binder in a weight ratio of 70:15:15 in NMP solvent. The resulting slurry was uniformly coated onto a copper foil current collector and dried at 100 °C under vacuum for 12 h. The dried electrode was cut into circular disks with widths of 12 mm. The mass loading and thickness of anode electrode material were ~0.7 to ~1.0 mg and 10 μm, respectively. Li metal foil was used as the counter/reference electrode and a Celgard membrane was used as the separator. The electrolyte was 1 M LiPF_6_ in a mixture of ethylene carbonate and diethyl carbonate (EC:DEC, 1:1 *v*/*v*). Galvanostatic charge–discharge (GCD) tests were performed within a voltage window of 0.01–3.0 V vs. Li^+^/Li using a battery analyzer (LAND CT2001A). Cyclic voltammetry (CV) was carried out on a potentiostat (CH1660E) electrochemical workstation at a scan rate of 0.2 mV s^−1^. Electrochemical impedance spectroscopy (EIS) measurements were conducted over a frequency range from 100 kHz to 0.01 Hz with an AC amplitude of 5 mV. The specific capacities reported in this study are calculated based on the total mass of the active composite material (CoPc + CNT) in the electrode.

## 3. Results and Discussion

### 3.1. Material Synthesis and Structural Characterization

**Figure 1:**
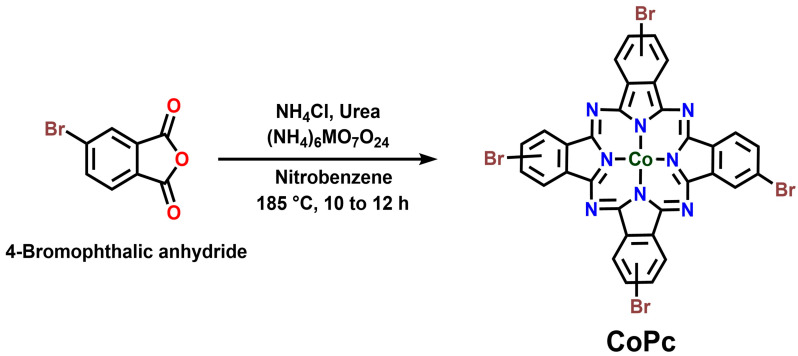
Schematic route of CoPc complex.

**Figure 2:**
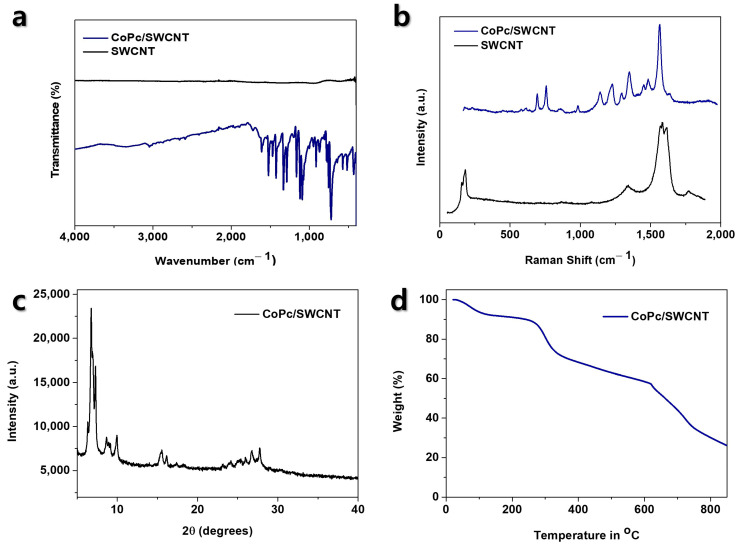
Characterization of CoPc/SWCNT composites: (**a**) FT-IR spectra of SWCNT and CoPc/SWCNT composite; (**b**) Raman spectra of SWCNT and CoPc/SWCNT composites; (**c**) PXRD patterns of CoPc/SWCNT; and (**d**) TGA thermogram of CoPc/SWCNT complex.

The synthetic strategy for the CoPc-CNT hybrids is illustrated in Figure 1. CoPc was first synthesized via a one-pot condensation reaction, with ammonium molybdate serving as an efficient catalyst. The subsequent hybrid formation was achieved through a non-covalent approach, where the planar CoPc molecules spontaneously anchor onto the graphitic surface of the CNTs via strong π-π interactions in NMP, a solvent known for its excellent dispersibility for both CNT and MPcs. The successful formation of CoPc and their interaction with CNTs were confirmed by FT-IR and Raman spectroscopy. Figure 2a shows the FT-IR spectra of pristine SWCNTs and CoPc/SWCNT. The absence of significant sharp intense absorption peaks in the pristine SWCNT confirms the samples’ purity and minimal presence of oxygenated functional groups. CoPc/SWCNT shows characteristic peaks at ~1608 cm^−1^ (C=C/C=N stretching of the isoindole units) and ~1285 cm^−1^ (C-N stretching). Raman spectroscopy (Figure 2b) provided definitive evidence for the successful non-covalent functionalization and strong electronic coupling between CoPc and the nanotube surface. The spectrum of pristine SWCNTs displays characteristic peaks: the D-band (~1336 cm^−1^), related to structural defects, the G-band (~1586 cm^−1^), corresponding to the in-plane vibration of sp^2^-hybridized carbon, and the radial breathing modes in the low-frequency region. Upon formation of the CoPc/SWCNT hybrid, three key changes are observed: first, an upshift in the G-band position, indicating charge transfer from the CNT to the CoPc molecule; second, an increase in the D/G band intensity ratio, suggesting an enhanced defect density due to the π-π stacking interaction that disrupts the sp^2^ carbon network; and finally, the emergence of new peaks corresponding to the vibrational modes of the CoPc macrocycle. Collectively, these spectral shifts and changes confirm a strong, non-covalent interaction that modifies the electronic environment of the SWCNTs, which is crucial for enhancing charge transfer in the electrochemical application.

The X-ray diffraction (XRD) analysis was employed to investigate the crystallographic structure of the synthesized materials (Figure 2c). The pattern for CoPc/SWCNT exhibits characteristic diffraction peaks, notably a strong reflection at a low angle (~7°) and confirms the successful formation of the crystalline Pc macrocycle. Thermogravimetric analysis (TGA) was employed under an air atmosphere to quantify the composition of the CoPc/SWCNT hybrid and confirm its thermal stability (Figure 2d). In contrast, the CoPc/SWCNT hybrid exhibits a distinct two-step decomposition process. The first major weight loss step in the range of 250–350 °C is attributed to the combustion and pyrolysis of the organic CoPc component. The second step, occurring above 500 °C, corresponds to the subsequent combustion of the SWCNT scaffold. The mass ratio between these two decomposition steps allows for an estimation of the approximate loading of CoPc within the hybrid material. By calculating the percentage of total mass lost in this first decomposition step relative to the initial mass, the mass loading of the active CoPc material was estimated to be approximately 30%. This confirms the successful formation of the hybrid with a significant fraction of the electroactive species.

**Figure 3:**
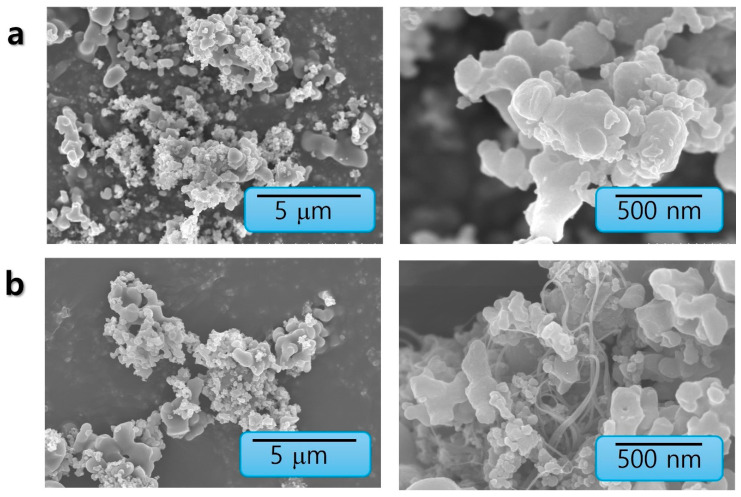
SEM images of (**a**) CoPc and (**b**) CoPc/SWCNT.

**Figure 4:**
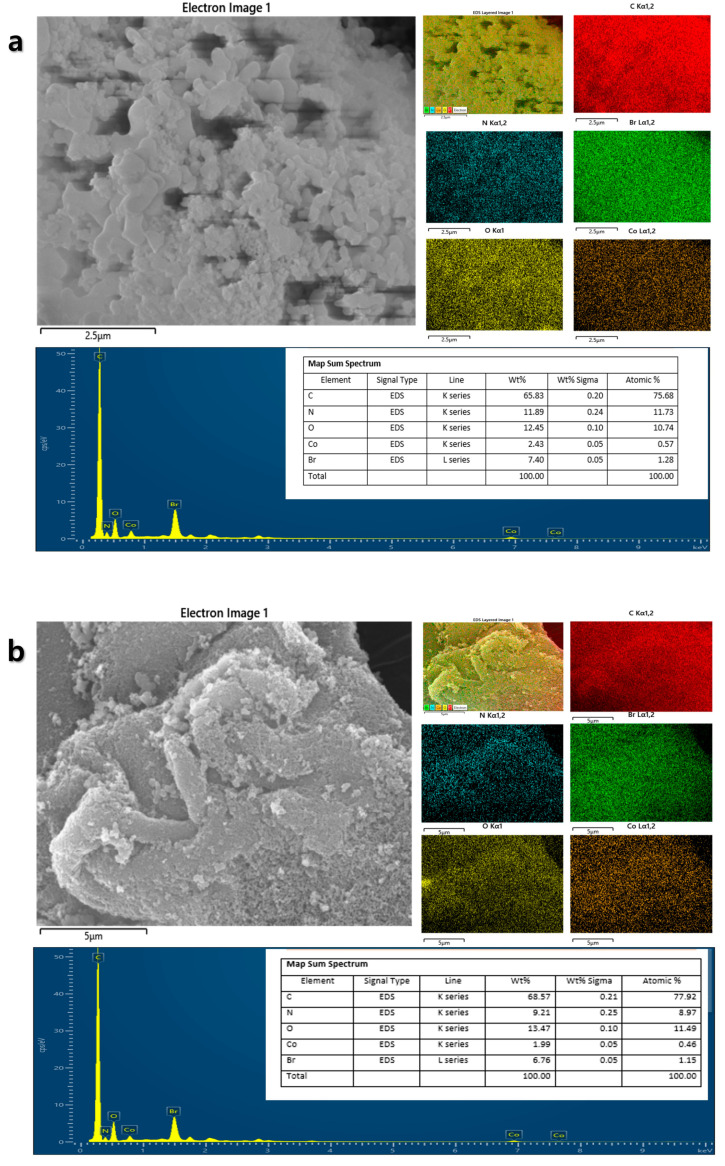
EDX results and elemental mapping image for (**a**) CoPc and (**b**) CoPc/SWCNT.

**Figure 5:**
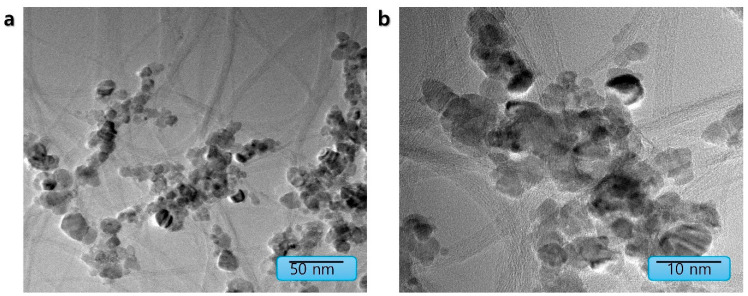
TEM images of (**a**) CoPc/SWCNT at 10 nm range and (**b**) CoPc/SWCNT at 50 nm range.

Figure 3 and Figure 4 present the morphological analysis using Scanning Electron Microscopy (SEM) coupled with Energy-Dispersive X-ray Spectroscopy (EDS) provided direct morphological and elemental evidence for the successful formation of the CoPc/SWCNT hybrid. SEM imaging revealed a stark contrast between the pristine CoPc, which appeared as large, irregular microcrystalline aggregates, and the CoPc/SWCNT composite, which exhibited a uniform, web-like morphology where the SWCNT bundles were thoroughly wrapped and interconnected by a coating of CoPc. EDS elemental mapping of pristine CoPc primarily consisted of C, N, O, Co, and Br with mass percentages of 75.68%, 11.73%, 10.74%, 0.57%, and 1.28%. Similarly, the hybrid structure confirmed the homogeneous distribution of C, N, O, Co, and Br with mass percentages of 77.92%, 8.97%, 11.49%, 0.46%, and 1.15%, respectively, provided definitive proof that the CoPc was successfully anchored onto the nanotubes, rather than simply being physically mixed. To elucidate the microstructure and successful formation of the hybrid material, transmission electron microscopy (TEM) was employed (Figure 5). TEM images of CoPc/SWCNT composite, showing its morphology at two different scales, such as 50 nm and 10 nm range, were taken. The images provide direct visual evidence of the presence and uniform coating of the SWCNT by the CoPc molecule. The conformal layer attributed to the strong interaction between Pc macrocycles and the CNT walls is crucial for the observed electrochemical enhancement. This intimate contact facilitates rapid electron transfer behavior of the coating and maximizes the accessibility of active sites for Li-ion coordination and storage. This unique architecture directly underpins the hybrid’s exceptional capacity and stability.

**Figure 6:**
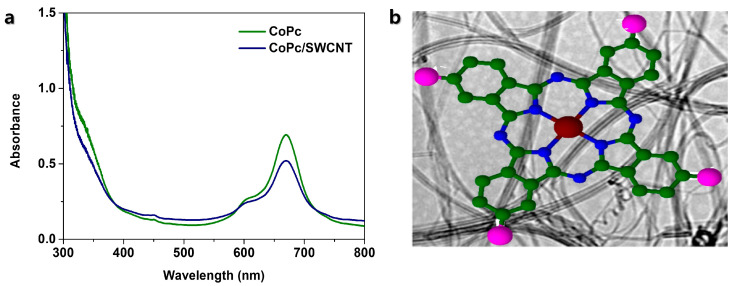
(**a**) UV-Vis spectra of both pristine CoPc and CoPc/SWCNT composite; (**b**) Structure of CoPc/SWCNT composite.

Figure 6a shows the UV-Vis spectra of both pristine CoPc and CoPc/SWCNT complexes. The characteristic absorption spectrum of CoPc reveals two primary electronic transitions with the B-band at 340 nm attributed to the n-π* transition and Q-band at 670 by the π-π* transition, which is responsible for the intense blue-green color of Pc [24,25]. The formation of the CoPc/SWCNT composite induces significant changes in this spectrum. The slight blue shift in the B-band from 340 nm to 324 nm strongly indicates a π-π stacking interaction between the conjugated macrocycle of CoPc and the sp^2^-hybridized surface of SWCNT. Notably, the position of the Q-band remains unchanged at 670 nm, which is a critical observation. It confirms that the central Co ions’ coordination geometry and electronic state are preserved upon attachment to the SWCNT. This means the primary function of the cobalt center is its ability to undergo redox cycling between Co^2+^ and Co^+^ during the lithiation/delithiation process. Figure 6b shows the structural elucidation of CoPc and SWCNT composite via π-π stacking interactions. Overall, Figure 6 confirms a successful functionalization of CoPc and SWCNT via π-π stacking, which facilitates electrical conductivity without compromising the intrinsic electrochemical activity of the Co^2+^/Co^+^ redox couple.

### 3.2. Electrochemical Performance as Li-Ion Battery Anodes

**Figure 7:**
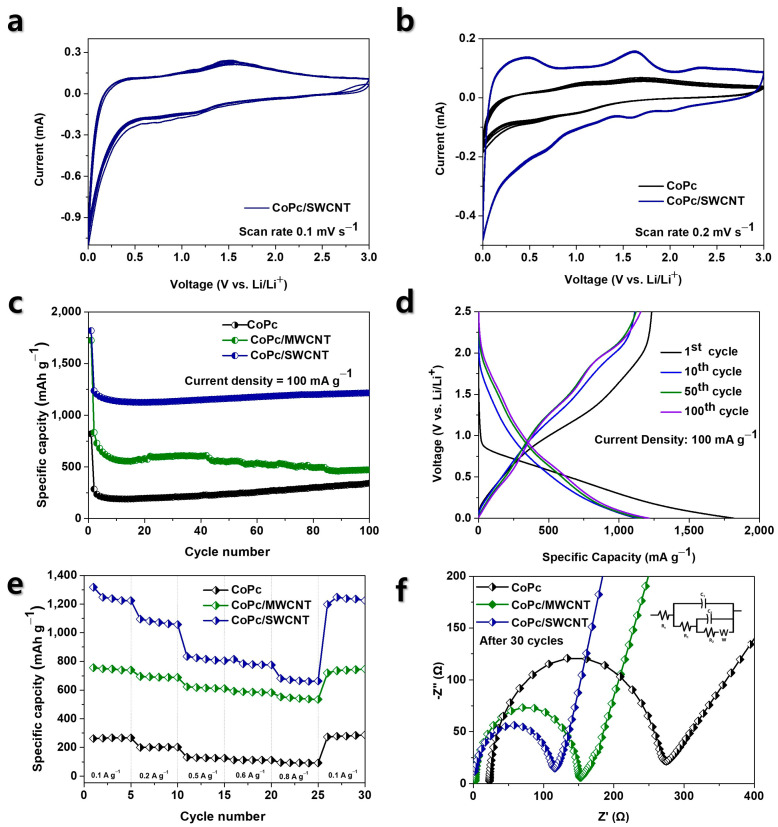
(**a**) CV curves of CoPc/SWCNT electrode at a scan rate of 0.1 mV s^−1^ in the initial three cycles; (**b**) CV curves of CoPc and CoPc/SWCNT electrodes at a scan rate of 0.2 mV s^−1^ in the initial three cycles; (**c**) Cycling performances of CoPc, CoPc/MWCNT, and CoPc/SWCNT at 0.1 A g^−1^; (**d**) Charge–discharge profile for CoPc/SWCNT; (**e**) Rate capacity of all three electrodes at different current densities; (**f**) EIS Nyquist plots after 30 cycles for CoPc, CoPc/MWCNT, and CoPc/SWCNT, and inset shows the equivalent circuit used to fit the EIS data.

The electrochemical performance of the pristine CoPc, CoPc/MWCNT, and CoPc/SWCNT electrodes was systematically evaluated. Figure 7a displays the CV curves of the CoPc/SWCNT hybrid electrode at a scan rate of 0.1 mV s^−1^ over the initial three cycles to access the evaluation of its redox peaks and evaluate the activation process and cycling stability. To directly compare performance, Figure 7b presents the CV profile of both the pristine CoPc and the CoPc/SWCNT composite at a scan rate of 0.2 for their first three cycles [26]. In Figure 7b, the CV reveals three main pairs of redox peaks at approximately 0.42/0.66 V, 1.62/1.56 V, and 2.32/2.02 V (vs. Li/Li^+^). The first pair, observed predominantly in the initial cycle, is attributed to irreversible processes including the formation of the solid electrolyte interphase (SEI) and the initial reduction in the cobalt center. The two subsequent pairs are highly reversible and are the primary contributors to the capacity. The prominent peaks centered around 1.6 V are assigned to the reversible redox reaction of the Co^2+^/Co^+^ couple [27]. The pair at higher potential, around 2.2 V, is associated with multi-electron redox processes occurring within the conjugated Pc ligand itself. The excellent reversibility and stability of these latter two pairs confirm the successful integration of CoPc with SWCNTs, which enhances the structural integrity and facilities efficient electron transfer during lithiation/delithiation.

The long-term cycling performance and electrochemical characters of the prepared anodes were critically evaluated. As depicted in Figure 7c, a stark contrast in cycling stability is observed among the three materials. The pristine CoPc electrode suffers from increased capacity, retaining only 327 mAh g^−1^ after 100 cycles, a consequence of its inherent low electrical conductivity and dissolution of active materials into the electrolyte. Functionalization with MWCNTs mitigates these issues, yielding a significantly improved and stable capacity of 488 mAh g^−1^. However, the CoPc/SWCNT hybrid demonstrates exceptional performance, achieving the highest reversible capacity of 1216 mAh g^−1^ after 100 cycles at 0.1 A g^−1^ with outstanding stability. The GCD profile of the CoPc/SWCNT electrode (Figure 7d) provides further insight into its electrochemical behavior. The profiles for the 1st, 10th, 50^th^, and 100th cycles exhibit clear voltage plateaus that align perfectly with the reversible redox peaks observed in the CV analysis, confirming the Faradaic reactions associated with the cobalt center and the organic ligand. Table 1 provides a direct performance comparison of the three anode electrode materials, evaluating their initial and 1st cycle discharge capacities. And Table 2 shows the coulombic efficiency (CE) over the first 10 cycles to assess the reversibility of the Li storage reaction. The initial discharge and charge capacity are 1823 mAh g^−1^ and 1280 mAh g^−1^ respectively, resulting in an initial coulombic efficiency (ICE) of 70.21%. This initial capacity loss is typical and it is primarily attributed to the irreversible formation of the SEI. Crucially the remarkable overlap of the subsequent profiles and the maintenance of a high reversible capacity underscore the excellent electrochemical reversibility and structural integrity of the CoPc/SWCNT hybrid anode over extended cycling.

The superior electrochemical kinetics of the CoPc/SWCNT hybrid were qualitatively confirmed through rate capability tests and EIS. Table 3 and Table 4 provide complementary experimental data to evaluate the electrode kinetics and high-rate performance. The CoPc/SWCNT electrode delivers exceptional capacities of 1237, 1068, 816, 775, and 666 mAh g^−1^ at progressively higher current densities from 0.1 to 0.8 A g^−1^. This outstanding performance starkly surpasses that of the CoPc/MWCNT and pristine CoPc anodes, which suffer from more significant capacity decay due to slower reaction kinetics [9,10]. When the current density is returned to 0.1 A g^−1^, the CoPc/SWCNT hybrid recovers nearly its original capacity (99.4%), demonstrating exceptional structure resilience and electrochemical reversibility even after high-rate cycling (Figure 7e). The EIS data and corresponding equivalent circuit fitting provide a direct physical relationship between these enhanced performances (Figure 7f). The Nyquist plots reveal a dramatically smaller semicircle for the CoPc/SWCNT hybrid, which corresponds to a very low total resistance. The exceptionally low solution resistance (Rₛ = 0.0013 Ω) indicates excellent intrinsic conductivity of the electrode composite [28,29]. More importantly, the significantly reduced charge transfer resistance (R_2_ = 88.7 Ω for CoPc/SWCNT vs. 108.5 Ω for CoPc/MWCNT and 237.4 Ω for pristine CoPc) confirms that the integration with SWCNTs creates a highly efficient pathway for electron transfer and ion diffusion at the electrode–electrolyte interface. This facilitated charge transfer kinetics is the fundamental reason behind the hybrid superior rate capability and high-power performance.

Generally, the SWCNT possesses higher intrinsic electrical conductivity, larger surface area, and more uniform surface compared to MWCNT [30,31]. These characteristics enable stronger π-π interactions and more efficient charge transfer pathway between the CoPc molecules and the CNT farmwork. The well aligned and defect-free structure of SWCNT facilitates rapid electron transfer and improved Li-ion diffusion, while their high specific surface area provides abundant electrochemically active sites for Li-ion storage. Consequently, the superior performance of CoPc/SWCNT hybrid stems from a powerful synergy between its components. The highly conductive SWCNT creates an electron expressway around the CoPc molecules, overcoming their innate insulating nature. This integration also ensures an effective dispersion of both materials, exposing more of the CoPcs active site for Li-ion reaction. Furthermore, the flexible SWCNT network as a mechanical scaffold buffers volume changes during cycling to preserve the electrode’s structure. This combination results in a complementary charge storage system where the CoPc contributes high redox capacity and the SWCNTs provide both conductivity and additional capacitance. Crucially, the use of SWCNTs is key, as their superior surface area and conductivity enable more efficient interaction with the CoPc than MWCNTs, leading to a significantly greater performance enhancement.

## 4. Conclusions

In summary, we have successfully synthesized a high-performance anode material for Li-ion batteries through the non-covalent functionalization of SWCNTs with CoPc. A combination of spectroscopic and microscopic techniques confirmed the successful formation of the hybrid via π-π stacking interactions, which resulted in a uniform coating of CoPc on the SWCNT surface. When evaluated electrochemically, the CoPc/SWCNT hybrid demonstrated exceptional performance, delivering a high reversible capacity of 1216 mAh g^−1^ after 100 cycles at 0.1 A g^−1^, significantly outperforming both pristine CoPc and a CoPc/MWCNT composite. Furthermore, the hybrid exhibited remarkable long-term cyclability, and superior rate capability. This enhanced performance is attributed to the synergistic combination of the high Li-ion storage capacity of the molecular CoPc and the superior electrical conductivity and mechanical stability provided by the SWCNT network, which facilitates rapid electron transport and mitigates active material dissolution. This work underscores the great potential of molecular-carbon nanomaterial hybrids and provides a simple, effective strategy for developing advanced organometallic-based electrodes for next-generation energy storage devices.

## Figures and Tables

**Table 1:** Performance comparison of three anode materials: CoPc, CoPc/MWCNT, and CoPc/SWCNT.

Materials	Initial Discharge Capacity (mAh g^−1^)	1 st Discharge Capacity. (mAh g^−1^)	Capacity Retention After 100 Cycles (%)
CoPc	821	290	39.82
CoPc/MWCNT	1729	834	28.22
CoPc/SWCNT	1823	1240	66.70

**Table 2:** Coulombic efficiency of the CoPc, CoPc/MWCNT, and CoPc/SWCNT electrodes at current density of 0.1 A g^−1^ for initial 10 cycles.

Cycle Number	Coulombic Efficiency (%)		
	CoPc	CoPc/MWCNT	CoPc/SWCNT
1st	50.93	65.93	70.21
2nd	73.46	84.16	97.64
3rd	82.67	89.64	98.56
4th	86.58	92.17	99.00
5th	88.53	93.86	99.24
6th	89.05	95.08	99.34
7th	90.15	95.89	99.45
8th	91.25	96.81	99.53
9th	91.56	97.27	99.57
10th	91.89	97.76	99.60

**Table 3:** Comparison of the rate capacity of the CoPc, CoPc/MWCNT, and CoPc/SWCNT electrodes.

Rate Capacity [mAh g^−1^]	0.1 A g^−1^	0.2 A g^−1^	0.5 A g^−1^	0.6 A g^−1^	0.8 A g^−1^	0.1 A g^−1^	Regained Capacity (%)
CoPc	269	201	123	107	89	270	100.3
CoPc/MWCNT	746	687	620	589	542	743	99.5
CoPc/SWCNT	1237	1068	816	775	666	1230	99.4

**Table 4:** Equivalent circuit parameters for the modified electrodes.

Electrode	R_s_ (Ω)	R_1_ (Ω)	R_2_ (Ω)	C_1_ (F)	C_2_ (F)	W (Ω)
CoPc	22.82	237.4	0.01	7.297 × 10^−8^	66.96	0.001183
CoPc/MWCNT	2.578	108.5	146	1.517 × 10^−7^	5.922 × 10^−8^	0.004626
CoPc/SWCNT	0.0013	88.7	1850	1.41 × 10^−7^	1.657 × 10^−6^	0.001617

## Data Availability

Data was created by own experiments and analysis and is available on request.
